# Vessel wall imaging: a promising enhancement in the management of inflammatory intracranial vasculopathy

**DOI:** 10.1093/braincomms/fcac226

**Published:** 2022-09-05

**Authors:** Dixon Yang, Mitchell S V Elkind

**Affiliations:** Department of Neurology, Vagelos College of Physicians and Surgeons, Columbia University, New York, NY, USA; Department of Neurology, Vagelos College of Physicians and Surgeons, Columbia University, New York, NY, USA; Department of Epidemiology, Mailman School of Public Health, Columbia University, New York, NY, USA

## Abstract

This scientific commentary refers to ‘Vessel wall magnetic resonance and arterial spin labelling imaging in the management of presumed inflammatory intracranial arterial vasculopathy’, by Benjamin et al. (https://doi.org/10.1093/braincomms/fcac157).

**This scientific commentary refers to ‘Vessel wall magnetic resonance and arterial spin labelling imaging in the management of presumed inflammatory intracranial arterial vasculopathy’, by Benjamin et al. (https://doi.org/10.1093/braincomms/fcac157).**

Cryptogenic stroke accounts for one-third of ischaemic strokes.^1^ Non-atherosclerotic intracranial vasculopathy, which can include medium-large vessel intracranial vasculitis, may account for some otherwise unexplained strokes.^2^ Infection, autoimmunity, neoplasm, metabolic, and genetic conditions can cause intracranial vasculitis.^3^ But diagnosis of intracranial vasculitis poses significant challenges, especially in the context of acute stroke care, due to its perceived rarity, delay in diagnostic testing, and limited sensitivity despite significant risk of morbidity with the gold standard diagnostic tool, a brain biopsy.^3^ Current stroke practice, therefore, needs accurate and non-invasive testing methods in the diagnosis of intracranial vasculitis.

Intracranial vessel wall magnetic resonance (VW-MR), utilizing high-resolution contrast-enhanced T1-weighted black blood images, is an increasingly available modality for study and diagnosis of presumed inflammatory intracranial arterial vasculopathy,^4^ which refers to a vessel imaging abnormality consistent with vasculitis without definitive histopathological confirmation. Intracranial VW-MR can offer additional information beyond the vessel lumen, but there are still considerable knowledge gaps in its clinical application.^4^ Recently in *Brain Communications*, Benjamin *et al*.^5^ describe a longitudinal case series of 11 ischaemic stroke patients with suspected inflammatory intracranial vasculopathy who underwent serial VW-MR with arterial spin labelling (ASL) MR perfusion as part of multidisciplinary stroke care.^5^ The authors sought an optimized clinical protocol that integrates VW-MR with ASL for patients with suspected inflammatory intracranial arterial vasculopathy.

From 2017 to 2018, the investigators identified 11 patients who presented to the hyperacute stroke unit at University College London Hospitals National Health Service Foundation Trust, a major urban stroke referral system in London, England. The patients all underwent specialist adjudication and were determined to have as their most likely stroke aetiology medium-large vessel inflammatory intracranial vasculopathy with an absence of evidence for other conventional causes. After referral to a dedicated multidisciplinary stroke clinic, they underwent baseline VW-MR with ASL. If a patient had circumferential or tramline intracranial vessel wall enhancement,^6^ which the authors considered confirmatory of presumed inflammatory intracranial arterial vasculopathy, they then obtained VW-MR with ASL at 6 months and 1 year as part of their routine clinical care. In addition to standard stroke evaluation, each patient underwent cerebrospinal fluid (CSF) analysis and whole body ^18^F-fluoro-deoxyglucose-positron emission tomography (^18^F-FDG-PET) to evaluate for systemic vasculitis. Treatment was individualized. The authors included only patients aged 55 years or less to minimize confounding from concomitant atherosclerosis.

Two blinded neuroradiologists experienced in VW-MR and ASL independently evaluated serial imaging for: (i) tramline or circumferential vessel wall enhancement consistent with vasculitis, (ii) degree of stenosis, and (iii) cerebral perfusion. They compared 1-year with baseline imaging and recorded imaging outcomes as either improvement or no change/disease progression. In addition, the authors used CSF analysis and ^18^F-FDG-PET to better classify cases. The authors devised three categories of vasculopathy: (i) infective, defined as CSF evidence for a specific infection; (ii) radiological evidence of inflammation with supporting evidence on additional testing (Inflam+), defined as CSF negative for a specific infectious agent but positive for markers of inflammation or ^18^F-FDG-PET arterial reactivity/avidity; and (iii) radiological evidence of inflammation with no supporting evidence from additional testing (Inflam-), defined as CSF negative for infectious agent or inflammatory markers and no evidence of ^18^F-FDG-PET arterial reactivity/avidity.

Of the 11 included patients, the median age was 36 years, 8 of 11 (73%) were women, and all were alive after 1 year of follow-up. The authors classified two patients as infective, three as Inflam+, and six as Inflam-. In the infective group, both patients were women (aged 26 and 32 years) with history of immune dysfunction, and both had CSF evidence of herpesvirus but no CSF pleocytosis. Both had radiographic evidence of multifocal intracranial stenosis. Of the three patients classified as Inflam+, all were also women, though slightly older (median age 41 years). In an example vignette, one Inflam+ patient had a history of Takayasu arteritis with circumferential intracranial vessel wall enhancement and associated vessel dilation. Finally, the Inflam- group had a median age of 45 years, an even distribution of sex, and a minority with comorbid vascular risk factors or immune dysfunction.

Eight of 11 patients (73%) received immunosuppressive treatment lasting more than 4 weeks, which involved high dose corticosteroids at a minimum. There was no statistical difference in wall enhancement at 1 year between those treated with immunosuppression and those not treated [6 (86%) versus 4 (50%), *P* = 0.20], but there was improvement in ASL-determined cerebral perfusion at 1 year in the immunosuppression group when compared to the untreated group [6 (100%) versus 2 (40%), *P* = 0.03]. Only one patient (Inflam+) had a recurrent clinical event, which occurred while off immunosuppressants.

Notably, while all cases had concentric intracranial vessel wall enhancement, reflecting presumed inflammatory vasculopathy, none had evidence of abnormal brain ^18^F-FDG-PET activity and only two had CSF pleocytosis, suggesting an added sensitivity of VW-MR in the arsenal of diagnostic modalities for inflammatory intracranial vasculopathy. Consistent with this finding, another case series suggested added sensitivity of VW-MR as an alternative to conventional digital subtraction angiography in the diagnosis of primary angiitis of the central nervous system.^7^ A sensitive non-invasive technique may be especially beneficial when early antithrombotic therapy, administered as part of acute stroke care, delays invasive diagnostic testing, such as CSF analysis or biopsy. Further, the authors add that use of VW-MR in their clinical practice changed management by initiation or adjustment of immunosuppressive therapy in two cases in which inflammatory vasculopathy was not initially considered as a stroke aetiology: an infective vasculopathy presumed to be caused by herpes simplex virus 2 and a patient with Takayasu arteritis. Moreover, the authors were able to monitor treatment response and all patients were spared the necessity of a brain biopsy.

Limitations of the study stem from the case series design, which reflects the rarity of inflammatory vasculopathy. In addition, the small sample size limited power and precluded detailed statistical analysis. More fundamentally, the study involved some circularity in the selection of patients and interpretation of imaging results. Circumferential enhancement, for example, was considered to reflect inflammatory vasculopathy, while eccentric inflammation was thought to represent atherosclerosis. However, the pathological literature confirming the specificity of concentric enhancement as indicative of vasculitis as opposed to atherosclerosis is limited. The nosology of cerebral vasculitis is a muddy field with little hard evidence, including a range of different disorders that may have been wrongly attributed to inflammation in the past (Call-Fleming syndrome, reversible cerebral vasospasm syndrome, ‘benign cerebral vasculitis,’ etc.). In addition, inflammation also plays a role in atherosclerosis, the most common cause of cerebral vasculopathy;^8^ thus, evidence of inflammation should not necessarily be taken as a sign that atherosclerosis is absent, or that the underlying cause is a rare disorder such as vasculitis rather than a common one such as atherosclerosis. Age < 55 years itself, moreover, cannot be considered to exclude atherosclerosis, as young patients are increasingly observed to have vascular risk factors, such as obesity and diabetes, that were previously thought to be relevant only at older ages.^9^ Similarly, it is unclear why an injured artery, as in dissection, would not be expected to have evidence of inflammation on imaging. Thus, the underlying notion that VW-MR showing enhancement is a marker of a specific vasculopathy distinct from other known causes of stroke requires further pathological confirmation. As a relatively new tool, understanding of VW-MR is evolving and interpretation of findings may vary.^4^ Any advantages of VW-MR in patient management or outcomes in this clinical setting would need further study, as the authors suggest.

The present case series by Benjamin *et al.*,^5^ therefore, demonstrates the potential real-world application of VW-MR with ASL in clinical practice for the diagnosis and management of suspected inflammatory intracranial vasculopathy presenting as cryptogenic stroke. The investigators conclude that their case series supports further rigorous study of VW-MR with ASL in this population of ischaemic stroke patients, especially when a better understanding of the pathologic correlates of VW-MR findings may be needed. Where feasible, an optimized and standardized clinical protocol with advanced imaging may help better estimate epidemiologic characteristics of inflammatory intracranial vasculopathy in the community. Similarly, VW-MR is being studied in other possible cryptogenic stroke mechanisms such as symptomatic substenotic atherosclerotic plaque and occult arterial dissections.^10^ In summary, this longitudinal case series strengthens the promise of VW-MR and ASL in the management of inflammatory intracranial vasculopathies, a potentially underrecognized mechanism of cryptogenic stroke.

## Data Availability

Data sharing is not applicable to this article as no new data were created or analysed.
